# QuickSilver: A Phase II Study Using Magnetic Resonance Imaging Criteria to Identify “Good Prognosis” Rectal Cancer Patients Eligible for Primary Surgery

**DOI:** 10.2196/resprot.4151

**Published:** 2015-04-14

**Authors:** Rectal Cancer Alliance Of Canada (RCAC)

**Affiliations:** ^1^see Acknowledgements

**Keywords:** MRI, Stage II and Stage III rectal cancer, primary surgery

## Abstract

**Background:**

Recently, two nonrandomized, prospective cohort studies used magnetic resonance imaging (MRI) to assess the circumferential resection margin to identify “good prognosis” rectal tumors eligible for primary surgery and have reported favorable outcomes.

**Objective:**

The objective of this project was to conduct a Phase II trial to assess the safety and feasibility of MRI criteria to identify “good prognosis” rectal tumors eligible for primary surgery in the North American setting.

**Methods:**

Patients with newly diagnosed primary rectal cancer attending surgical clinics at participating centers will be invited to participate in the study. The inclusion criteria for the study are: (1) diagnosis of rectal cancer (0-15 cm) from the anal verge on endoscopy and proximal extent of tumor at or below the sacral promontory on computed tomography (CT) or MRI; (2) meets all MRI criteria for “good prognosis” rectal tumor as defined by the study protocol; (3) 18 years or older; and (4) able to provide written consent. The initial assessment will include: (1) clinical and endoscopic examination of the primary tumor; (2) CT chest, abdomen, and pelvis; and (3) pelvic MRI. All potentially eligible cases will be presented at a multidisciplinary cancer conference to assess for eligibility based on the MRI criteria for “good prognosis” tumor which include: (1) predicted circumferential resection margin (CRM) > 1 mm; (2) definite T2, T2/early T3, or definite T3 tumor with < 5 mm of extramural depth of invasion (EMD); (3) any N0, N1, or N2; and (4) absence of extramural venous invasion (EMVI). All patients fulfilling the MRI criteria for “good prognosis” rectal cancer and the inclusion and exclusion criteria will be invited to participate in the study and proceed to primary surgery. The safety of the MRI criteria will be evaluated by assessing the positive CRM rate and is the primary outcome for the study.

**Results:**

We expect to have a minimum of 300 potentially eligible patients, and based on a 30% eligibility rate and 80% participation rate, it is expected that 75 patients will be recruited over the two year study period. A Data Safety Monitoring Committee has been organized, and the study will be stopped if a positive CRM of >10% is reported at any interim assessment, which will occur after every 25 patients accrued in the study.

**Conclusions:**

It is expected that the results of this study will show that use of MRI criteria to identify “good prognosis” rectal cancers eligible for primary surgery will be safe (ie, positive margin less than 10%). Therefore, these results will have significant potential to change the current management of rectal cancer in North America and result in improved quality of life for rectal cancer patients and survivors, while reducing overall health care costs.

**Trial Registration:**

ISRCTN05107772; http://www.controlled-trials.com/ISRCTN05107772/ (Archived by WebCite at http://www.webcitation/6WhhUhXkA).

## Introduction

### Preoperative Chemoradiotherapy for Stage II and III Rectal Cancer

Preoperative chemoradiotherapy (preCRT) is recommended for Stage II and III rectal cancer based on several, well designed randomized controlled trials (RCTs) that have shown preCRT significantly reduces the risk of local recurrence (LR) from 15% to 7.5% at 2 years [1-6]. Unfortunately, while preCRT reduces the risk of LR, it does not improve survival, leads to significantly poorer bowel and sexual function, and increases the risk of developing second malignancies compared to surgery alone [7-11]. Therefore, new approaches to improve selection and limit preCRT to Stage II and Stage III rectal cancer patients who are most likely to benefit from preCRT are important to improve the long term functional results and overall quality of life for rectal cancer patients, provided optimal oncologic outcomes can be obtained.

There are two recent, nonrandomized, prospective cohort studies (United Kingdom, MERCURY, and Germany) that have used magnetic resonance imaging (MRI) to assess the predicted circumferential resection margin (CRM) to identify “good prognosis” rectal tumors eligible for primary surgery [12,13]. The MRI criteria used for each of these studies are shown in Table 1. In these studies, patients with a MRI predicted “good prognosis” tumor underwent primary surgery, and the results showed favorable clinical outcomes with low rates of positive CRMs (3.3%, 4/122; 6.0%, 11/181) and 2 year LR (3.3%, 4/122), respectively.

### Objective of the Study

Therefore, the objective of this study is to conduct a Phase II trial to assess the safety of MRI criteria to identify “good prognosis” Stage II and Stage III tumors eligible for primary surgery in the North American setting.

**Table 1:** MRI criteria for “good prognosis” rectal cancer tumors eligible for primary surgery.

	United Kingdom (Mercury)	German
Predicted CRM	CRM > 1 mm	CRM > 1 mm
T^a^-category and EMD^b^	T1, T2, or T3 with ≤ 5 mm EMD^b^	T1, T2, or any T3
N^c^-category	N0, N1, N2	N0, N1, N2
EMVI^d^	EMVI^d^ negative	Not assessed
Tumor height	Tumors 5 to 15 cm from the anal verge Tumors < 5 cm from anal verge with no invasion of the intersphincteric plane	Tumors 6 to 12 cm from the anal verge

^a^ T=primary tumor

^b^ EMD = extramural depth of invasion

^c^ N=regional lymph nodes

^d^ EMVI = extramural venous invasion

### Study Overview

This is a 2 year Phase II study to evaluate the safety of MRI criteria to identify “good prognosis” Stage II and Stage III rectal cancer eligible for primary surgery. The safety of the MRI criteria will be evaluated by assessing the positive CRM rate in this “good prognosis” subset of the Stage II and Stage III rectal cancer patients. The MRI criteria will be considered safe if a positive CRM rate of less than 10% is achieved.

## Methods

### Start-Up Period, 0-3 Months

Research Ethics Board approval and data sharing agreements have been obtained at the lead and participating sites for the study. The project will be launched via radiology, surgery, and pathology webinars with all participating physicians (at all sites) to review the study protocol and data collection processes and complete relevant training sets.

### Patient Sample and Recruitment, 3-21 Months

Newly diagnosed rectal cancer patients attending surgical clinics at participating centers will be invited to participate in the study.

The *inclusion criteria* for the study are: (1) diagnosis of rectal cancer (0-15 cm) from the anal verge on endoscopy and proximal extent of tumor at or below the sacral promontory on computed tomography (CT) or MRI; (2) meets all MRI criteria for “good prognosis” rectal tumor as defined by study protocol (see Table 2); (3) 18 years or older; and (4) able to provide written consent.

The *exclusion criteria* for the study are: (1) planned abdomino-perineal resection (APR) based on pretreatment assessment; (2) planned local excision based on pretreatment assessment; (3) T1/early (primary) T2 tumor on preoperative imaging (MRI and/or transrectal ultrasound); (4) suspicious extramesorectal lymph nodes on MRI; (5) unable to undergo MRI due to contraindications (ie, claustrophobia, metal fragments, implanted metal devices); (6) metastatic disease (including extramesorectal lymph nodes, carcinomatosis, liver, lung); (7) pregnancy; (8) inflammatory bowel disease; (9) previous pelvic radiation; and (10) more than one primary tumor.

### Clinical Assessment

**Figure 1:**
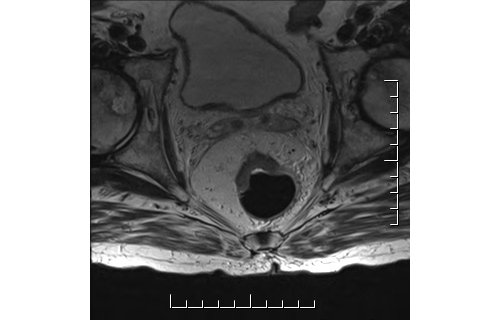
Mid rectal T3 tumor < 5 mm EMD and predicted CRM > 1mm. No suspicious lymph nodes and no EMVI. T: primary tumor; EMD: extramural depth of invasion; CRM: circumferential resection margin; and EMVI: extramural venous invasion.

A participating surgeon at each center will perform the initial clinical assessment. The surgeon will be responsible for facilitating the standard preoperative assessment that includes: (1) clinical and endoscopic examination of the primary tumor; (2) CT scan of chest, abdomen, and pelvis; and (3) pelvic MRI. The participating surgeon will be responsible for presenting all potentially eligible rectal cancer cases at a multidisciplinary cancer conference (MCC), which must be attended by the surgeon and at least one radiologist and radiation oncologist affiliated with the participating center. Alternatively, if a MCC is not available, the surgeon will be responsible for organizing a multidisciplinary meeting with Radiology and Radiation Oncology Site Leads. At MCC (or multidisciplinary meeting), all patients fulfilling the MRI criteria for “good prognosis” rectal cancer (Table 2) and the inclusion and exclusion criteria will be invited to participate in the study. Figure 1 shows an example of a MRI with a good prognosis tumor. After obtaining consent, this group of patients will proceed to primary surgery.

**Table 2:** MRI criteria for “good prognosis” and “poor prognosis” rectal tumors.

MRI criteria	Good prognosis	Poor prognosis
Predicted CRM	>1 mm (nonthreatened)	≤ 1 mm (threatened)
T-category^a^ and EMD^b^	Definite T2, T2/early T3, or definite T3 with EMD^b^< 5 mm	Definite T3 with EMD^b^ > 5 mm or T4
N^c^-category	Any N0, N1, or N2	Any N0, N1, or N2
EMVI^d^	Absent or equivocal	Present

^a^ Includes, primary tumor, discontinuous tumor nodes, suspicious lymph nodes, and extramural venous invasion; definite T1 and T1/early T2 tumors will be excluded from study protocol

^b^ EMD = extramural depth of invasion

^c^ N=regional lymph nodes

^d^ EMVI = extramural venous invasion

### Radiologic Assessment

Each MRI will be reported according to the standard protocol for the study [14]. At minimum, the MRI protocol must include high resolution, axial oblique T2 weighted sequences. The MRI report must include: (1) distance to the mesorectal fascia (predicted CRM), (2) T-category (includes, primary tumor, discontinuous tumor nodes, suspicious lymph nodes, and extramural venous invasion; definite T1 and T1/early T2 tumors will be excluded from study protocol) including extramural depth of invasion into the mesorectum (EMD) for all tumors T3 or greater, (3) absence or presence of suspicious lymph nodes, and (4) absence or presence of extramural venous invasion (EMVI). Although presence of suspicious lymph nodes is not a MRI criterion for “good prognosis” tumors, this information will be recorded so that we will be able to assess the accuracy of lymph node assessment on MRI compared to the final pathology since all patients are undergoing primary surgery. If there is any uncertainty regarding these MRI criteria, the reporting radiologist will be instructed to review the MRI with the Site Lead Radiologist to achieve consensus. If consensus is not achieved and/or uncertainty still exists after review by the Site Lead, the reporting radiologist will be asked to contact the Lead Radiologists (LM, MF) for the study for central review. The central study office (SS, EK) will review the MRI reports to ensure that all of these MRI criteria are reported. In the case of missing data, the Radiology Site Lead will be contacted to obtain this data. Participating centers and radiologists will be encouraged to use a synoptic MRI template for the study; however, this is not mandatory for participation in the study [15]. Prior to the start of the study, a Radiology Webinar will be organized to review MRI protocol, definitions, and interpretation of MRI criteria, and educational materials will be provided. In addition, Radiology training sets will be developed and will be required to be successfully completed by participating radiologists.

### Surgical Assessment

The surgical procedure will be left to the discretion of the surgeon and will involve a partial mesorectal excision for upper rectal cancers (above the anterior peritoneal reflection) and total mesorectal excision (TME) for mid and low rectal cancers (below the anterior peritoneal reflection) [16]. To be eligible for the study, surgeons must have completed colorectal or surgical oncology fellowship training in Canada or the United States. Surgeons will also be encouraged to use a synoptic Operative Report template that has been pilot tested and is currently being used in British Columbia; however, this is not mandatory for participation in this study [17]. The central study office (SS, EK) will review the surgical reports to ensure that all of the surgical information required is reported. In the event that there is missing data, the treating surgeon and Site Lead will be contacted.

Surgery should occur as soon as possible from the time of decision for surgery. Prior to the start of the study, participating surgeons will be required to attend the Pathology Webinar in which the protocol for gross evaluation of the TME specimen will be reviewed and discussed. Participating surgeons will be provided with educational materials and will be encouraged to present cases with positive CRM or incomplete TME at MCC for feedback and audit from the site group.

### Pathologic Assessment

Each surgical specimen will be processed and reported according to the standard protocol by Quirke et al and must include both macroscopic (quality of the TME) and microscopic assessment (including T-category; EMD; EMVI; and N-category, regional lymph nodes) [18]. Photographs of the gross specimen and serial section are required. If there is any uncertainty about any of these criteria, the reporting pathologist will be instructed to have this reviewed by the Site Lead to achieve consensus. However, if consensus is not achieved or uncertainty still exists after review by the Site Lead, the Site Lead will be asked to contact the Lead Pathologists (RK, DD) for central review. The central study office (SS, EK) will review the pathology reports to ensure that all of the required criteria have been reported. In the case of missing data, the Pathology Site Lead will be contacted to obtain the missing data. Prior to the start of the study, a Pathology Webinar to review the Quirke method will be organized. At the webinar, there will be a review of the Quirke protocol and definitions and interpretation of the reported criteria. Educational materials will be provided and a Pathology training set will be developed and will be required to be successfully completed by participating pathologists. Participating pathologists will be encouraged to use the College of American Pathologists checklist, however, this is not mandatory for participation in the study [19].

### Recommended Follow-Up

**Figure 2:**
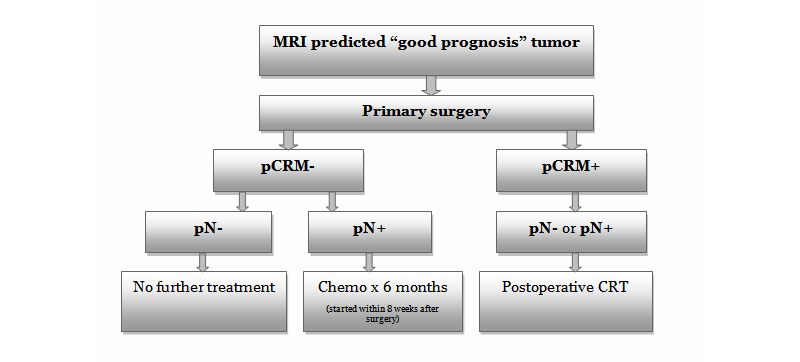
Recommended follow-up for trail participants. MRI: magnetic resonance imaging; pCRM-: negative circumferential resection margin; pCRM+: positive circumferential resection margin; pN-: lymph node negative; pN+: lymph node positive; CRT: chemoradiotherapy; and Chemo: chemotherapy. *No further treatment (bottom, left box): Chemotherapy may be considered at the discretion of the treating oncologist for CRM- and LN- patients for high-risk features such as extramural venous invasion (EMVI).

Figure 2 shows the recommended follow-up for the study. For patients with a negative CRM and no lymph node involvement, no further treatment will be recommended and these patients will be placed in a surveillance program as per the institutional protocol. However, chemotherapy may be considered in these patients if there are other high risk features such as EMVI present. Patients with a negative CRM and positive lymph nodes will be recommended to undergo adjuvant chemotherapy as per institutional protocols. It is preferred that these patients do not receive postoperative radiation, as the main objective of the study is to avoid radiotherapy, however, the final decision about postoperative radiation will be left to the discretion of the treating physicians. Patients with a positive CRM irrespective of lymph node status (ie, positive or negative lymph nodes) will be recommended to have postoperative chemoradiotherapy as per the institutional protocols. The follow-up for each study patient will be recorded.

### Data Collection

Participating surgeons will be required to send the MRI report, operative report, and pathology report to the central study office via facsimile (FAX). All patient documents will be assigned a unique identification number by the study coordinator and will be deidentified by the participating center. The study coordinator at the central office will send regular reminders and updates to all participating physicians and will ensure data collection is complete for each patient. A stand alone FAX machine will be kept in the study coordinator’s locked office and will be used only for the purposes of this study.

## Results

The primary outcome for the study is the positive CRM rate. A positive margin will be defined as any macroscopic or microscopic tumor, discontinuous tumor nodule, or a positive lymph node located within 1 mm of the CRM on final pathologic assessment. We have 30 high volume surgeons at 16 centers participating in this study who see a minimum of 10 new rectal cancer patients over the 18 month time period. Therefore, a minimum of 300 potentially eligible patients will be assessed, and based on the United Kingdom and German studies, it is estimated that 30% (n=90) will be eligible to participate in the study. Assuming an 80% participation rate, it is expected that 75 patients will be recruited over the two year study period.

If seventy-five patients participate, this will provide a 95% confidence interval half-width precision of 6.7% around a point estimate of 10% for the positive CRM rate. If the point estimate for the positive CRM rate is smaller than 10%, the precision around the point estimate will increase.

A Data Safety Monitoring Committee has been organized and will consist of the study statistician, one surgeon, one radiation oncologist, and one pathologist (who are not participating in the study). The study will be stopped if a positive CRM of >10% is reported at any interim assessment, which will occur after every 25 patients accrued in the study.

The secondary outcomes for the study include 2 year LR and disease free survival rates. Descriptive statistics will be used to report: (1) tumor characteristics, (2) positive CRM rate, (3) MRI findings (T-category, N-category, predicted CRM, EMVI), and (4) final pathology (quality of the TME, CRM, T-category, N-category, EMVI). Regression analysis will be performed to assess if any clinical variables are predictive of positive CRM. In addition, comparing the MRI and pathology findings and assessing interobserver agreement for each of the MRI criteria used to identify “good prognosis” tumors will evaluate the accuracy of the MRI interpretation.

This trial is currently recruiting patients.

## Discussion

### One Day Investigator’s Meeting

Our team organized a one day investigator’s meeting on June 28, 2013 in Toronto, Ontario, Canada. Colorectal surgeons (n=22), radiation oncologists (n=8), radiologists (n=4), and pathologists (n=1) from high volume rectal cancer centers across Canada attended the meeting. In addition, Dr Gina Brown, the principal investigator of the MERCURY trial attended the meeting. The overall objective of the meeting was to: (1) select MRI criteria to identify “good prognosis” rectal tumors (ie, tumors at low risk for LR) eligible for primary surgery, and (2) finalize a protocol to evaluate the safety of using these MRI criteria to select “good prognosis” tumors eligible for primary surgery. Prior to the meeting, the MERCURY and German trial papers, as well as a draft study protocol, were circulated to the participants.

At the meeting, Dr Brown gave a formal presentation of the MERCURY trial results, and the investigative team presented the German trial results. After each presentation, there was a moderated discussion in which the following “good prognosis” MRI criteria were discussed: (1) definition of a threatened CRM in millimeters, (2) T-category and EMD, (3) lymph node assessment, (4) height of tumor, and (5) EMVI.

For the meeting, the following definitions of terms were used. CRM refers to the MRI predicted distance to the mesorectal fascia (MRF). EMD is the extension of the tumor into the perirectal fat beyond the muscularis propria and applies to all T3 and T4 tumors. EMVI is a pathologic, microscopic feature that refers to invasion of large vessels deep to the muscularis propria and is an independent, negative prognostic factor of survival and can be accurately detected on MRI. The highlights of the group discussion for each MRI criteria are detailed below.

### Definition of a Threatened Circumferential Resection Margin

Both the MERCURY and German trial defined a threatened CRM on MRI as < 1 mm, since a CRM < 1 mm has been shown to significantly increase the risk of LR [20,21]. While the German trial defined a threatened CRM as < 1 mm to the primary tumor, discontinuous tumor deposit, EMVI, or suspicious lymph nodes, MERCURY did not include suspicious lymph nodes in their definition of threatened CRM. However, for both studies a positive pathologic margin was considered < 1 mm to the primary tumor, discontinuous tumor deposit, EMVI, or positive lymph nodes. In the MERCURY trial, the majority of the positive pathologic margins were due to the primary tumor. There is also some evidence from the Dutch trial that the LR rate from a positive margin due to a lymph node is significantly lower than a positive margin due to a primary tumor [20]. Overall, our group was concerned about the definition of a threatened margin as < 1 mm, as this was considered very little room for error, especially in a low, anterior tumor in a male pelvis. While an alternative definition of < 5 mm was proposed, the main concern with the use of this definition was that it would result in many more patients being ineligible for the study due to a threatened CRM, and significantly affect recruitment. Furthermore, since a large proportion of these patients would have a negative margin with primary surgery, our group felt that this definition would limit the generalizability of the study. At the end of the discussion, while all of the group members indicated they would not use the < 1 mm definition in their current clinical practice, the majority agreed that they would be willing to evaluate the safety of the < 1 mm definition within the context of the study protocol. The group also felt that use of the < 1 mm definition was important to validate the results of the MERCURY and German trials and the generalizability of this approach.

### Primary Tumor-Category and Extramural Depth of Invasion

The MERCURY trial considered T3 tumors with < 5 mm EMD as “good prognosis” tumors, while the German trial considered any T3 tumor as a “good prognosis” tumor. The rationale for the MERCURY trial definition was based on a population-based study in which T3 tumors were classified based on EMD [22]. This study reported similar LR and disease free survival rates between T2 tumors and T3 tumors with < 5 mm EMD. Based on these data, the MERCURY group conducted a prospective cohort study to assess the accuracy of EMD measured on MRI using the pathologic specimen as the gold standard in 295 rectal cancer specimens. The MERCURY investigators found a mean difference of only -0.05 mm (95% CI -0.49 mm to 0.40 mm) between MRI and pathologic measurements for EMD [14]. Based on this evidence and expert opinion, our group achieved consensus to consider patients with T3 rectal tumors with < 5 mm EMD as having “good prognosis” tumors [23].

While the MERCURY study included T1 tumors as “good prognosis”, the German trial excluded T1 tumors. Since all of the participating centers across Canada currently were not offering preCRT to T1 or T2 tumors, our group decided to exclude definite T1 and T1/early T2 tumors from the study protocol. However, due to the difficulty discriminating T2/early T3 tumors on MRI, our group decided to include T2/early T3 tumors, as we felt there was relatively high potential for these tumors to be understaged on MRI.

### Lymph Node Assessment

Both the MERCURY and German trials considered any N-category (N0, N1, N2) as “good prognosis” tumors. The rationale for this was that lymph node evaluation on MRI (as well as other imaging modalities) is relatively poor. Furthermore, the results of the MERCURY trial showed that lymph node involvement was not an independent predictor of LR or survival. This finding is particularly controversial since the small proportion of node positive cases (18.0%, 22/122) in the study does not provide enough power to strongly support this conclusion and all previous rectal cancer RCTs have shown lymph node involvement is a positive and independent predictor of LR. However, it is important to note that the preoperative staging in previous rectal cancer RCTs was primarily based on clinical examination, which has shown to be highly inaccurate and overstaging was reported in 20% of patients in the German trial undergoing preoperative staging with transrectal ultrasound [3,24]. Therefore, it may be that with more appropriate staging (with MRI) that lymph node involvement may not be as important a predictor of LR as previous RCTs have shown. While our group was very concerned about considering N1 and N2 disease as “good prognosis” tumors due to the limited and contradictory evidence, the group also agreed that this was one of the most critical issues to address in the study protocol. Therefore, while all of the group members indicated that they would not be willing to consider N1 and N2 disease as “good prognosis” tumors in their own practice, the majority agreed they would be willing to evaluate the safety of considering N1 and N2 disease as “good prognosis” within the context of a study protocol. The group also felt that considering N1 and N2 disease as “good prognosis” tumors would be important to validate the results of the MERCURY and German trials and the generalizability of this approach.

### Height of Tumor

The MERCURY trial included patients with tumors 0-15 cm from the anal verge and included low rectal cancers requiring APR, while the German trial included tumors > 6 cm and < 12 cm from the anal verge.

For our study, our group decided to include tumors 0-15 cm from the anal verge on endoscopy. However, in order to ensure that rectosigmoid tumors were not included in the study, we added an additional criterion that the proximal extent of the tumor had to be either at or below the sacral promontory on the sagittal sequence of the MRI.

Furthermore, the majority of participating centers indicated that their institutional protocol was to recommend preCRT to all patients with T2/early T3 tumors undergoing a planned APR. The rationale for this was the difficulty completing postoperative chemoradiation following APR, when a T2N0 MRI staged tumor is found to be T3 or node positive on final pathology. Therefore, our group decided to exclude patients with low rectal cancers requiring APR and include only patients for whom a restorative procedure was planned. In addition, patients undergoing a planned local excision were also excluded from the study.

### Extramural Venous Invasion

EMVI is a pathologic, microscopic feature that refers to invasion of large vessels deep to the muscularis propria and is a known independent prognostic indicator of distant recurrence and survival in rectal cancer [25]. In previous work, the MERCURY group developed a MRI-based classification for EMVI. Using this classification, the MERCURY group reported a 62% sensitivity and 88% specificity for MRI to detect EMVI using the pathologic specimen as the gold standard and reported fair interrater reliability for accurate detection of EMVI on MRI (kappa=.41, 95% CI 0.31-0.49) [26]. While univariate regression analysis showed that MRI detected EMVI was a negative predictor of recurrence free survival, this was not significant on multivariable regression analysis. While the group had some concern that EMVI was a relatively new MRI criterion for many radiologists, the group acknowledged that EMVI is most often found in the presence of other “poor prognosis” features and seldom the sole MRI criteria used to classify “good” and “poor” prognosis tumors. Therefore, similar to the other MRI criteria, the majority agreed to include MRI predicted EMVI within the context of the study protocol to validate the results of the MERCURY trial and assess the accuracy of MRI detected EMVI by comparing this result to final pathology. The group also agreed that an educational component and training session for participating radiologists be developed as part of the study protocol.

### Summary

Based on these discussions, the following MRI criteria were proposed by the group for “good prognosis” tumors: (1) rectal cancers 0-15 cm from anal verge with proximal extent at or below the sacral promontory on MRI and anterior resection (ie, restorative procedure) is planned; (2) distance to the MRF or predicted CRM > 1 mm (margin not threatened); (3) T2 and T3 tumors with < 5 mm EMD; (4) any N (N0, N1, or N2); and (5) EMVI absent.

A consensus vote on the proposed MRI criteria was conducted. The group voted anonymously using ballots, and the results of the vote were presented to the group. The group discussed the results of the vote, and revisions of the MRI criteria and subsequent rounds of voting were planned as necessary. The investigative team agreed a priori that consensus would be reached if 80% of the group voted “yes” to the proposed MRI criteria and had planned for 2 to 5 rounds of voting. However, after the first round of voting, 91% (31/34) of the participants voted “yes” to the proposed MRI criteria. The results were presented, and the three individuals who voted “no” identified themselves and explained the reasons for their vote. There were two of these individuals that were concerned about the definition of a threatened margin < 1 mm and would have preferred this to be < 5 mm, and the third individual was concerned about including N1 and N2 disease as “good prognosis” tumors. Since consensus had been achieved on the first round of voting, no subsequent rounds of voting were conducted.

This study is highly relevant, as it is expected that the results of this study will show that use of MRI criteria to identify “good prognosis” rectal cancers eligible for primary surgery will be safe (ie, positive margin rate less than 10%). Therefore, these results will have significant potential to change the current management of rectal cancer in Canada and result in improved quality of life for rectal cancer patients and survivors, while reducing overall health care costs. Furthermore, these results would provide the necessary data to determine if an international RCT to address this question would be feasible based on sample size, recruitment, and cost. Last, standardization of preoperative MRI imaging, surgical, and pathological assessment across centers of excellence in Canada will be important for reporting long term outcomes for this study (ie, 2 year survival and LR rates, quality of life), improving the quality of patient care across Canada, and facilitating participation in future clinical trials on both a national and international level.

## Abbreviations

APR: abdomino-perineal resection
CRM: circumferential resection margin
CT: computed tomography
EMD: extramural depth of invasion
EMVI: extramural venous invasion
FAX: facsimile
LR: local recurrence
MCC: multidisciplinary cancer conference
MRF: mesorectal fascia
MRI: magnetic resonance imaging
preCRT: preoperative chemoradiotherapy
RCTs: randomized controlled trials
TME: total mesorectal excision
